# Corroborating behavioral evidence for the interplay of representational richness and semantic control in semantic word processing

**DOI:** 10.1038/s41598-021-85711-7

**Published:** 2021-03-17

**Authors:** Laura Bechtold, Christian Bellebaum, Paul Hoffman, Marta Ghio

**Affiliations:** 1 Department of Biological Psychology, Institute for Experimental Psychology, Heinrich Heine University Düsseldorf, Düsseldorf, Germany; 2 School of Philosophy, Psychology and Language Sciences, University of Edinburgh, Edinburgh, UK; 3 CIMeC - Center for Mind/Brain Sciences, University of Trento, Trento, Italy

**Keywords:** Human behaviour, Cognitive neuroscience

## Abstract

This study aimed to replicate and validate concreteness and context effects on semantic word processing. In Experiment 1, we replicated the behavioral findings of Hoffman et al. (Cortex 63,250–266, https://doi.org/10.1016/j.cortex.2014.09.001, 2015) by applying their cueing paradigm with their original stimuli translated into German. We found concreteness and contextual cues to facilitate word processing in a semantic judgment task with 55 healthy adults. The two factors interacted in their effect on reaction times: abstract word processing profited more strongly from a contextual cue, while the concrete words’ processing advantage was reduced but still present. For accuracy, the descriptive pattern of results suggested an interaction, which was, however, not significant. In Experiment 2, we reformulated the contextual cues to avoid repetition of the to-be-processed word. In 83 healthy adults, the same pattern of results emerged, further validating the findings. Our corroborating evidence supports theories integrating representational richness and semantic control mechanisms as complementary mechanisms in semantic word processing.

## Introduction

Concrete words (e.g., butterfly, train) show a processing advantage compared to abstract words (e.g., wisdom, tolerance). This so-called *concreteness effect*^1,2^ (CE) emerged in tasks requiring recall, comprehension and recognition of concrete and abstract words^3,4^. Theoretical approaches explained this processing advantage as due to differences regarding the information comprised in the conceptual representations of concrete and abstract word meanings. According to the *dual-coding theory*^5^ (DCT) representations of abstract words are based only on verbal information, while those of concrete words additionally rely on sensorimotor information. The higher amount of information available for concrete word processing leads to the CE. Another seminal theory, the *context availability-model*^2^ (CAM), considers a reduced availability of contextual information for abstract vs. concrete words as decisive for the CE. The CAM originated from the finding that context availability for a given word (i.e., the ease of retrieving contextual information for a given word) was a better predictor for reaction times in reading and lexical decision tasks than word concreteness^2^ and that providing a similar amount of contextual information for concrete and abstract words cancelled out the CE^6^.

More recent findings casted doubt on the explanatory power of either of these two competing theoretical approaches. In a replication of one of the studies that motivated the development of the CAM^6^, context availability canceled out the CE only if subjective familiarity was not controlled^7^. Furthermore, studies controlling for imageability, context availability and/or familiarity reported an inverse CE, with faster and/or more accurate responses to abstract than concrete words^8–10^, which neither of the two theoretical approaches can explain. Instead, this processing facilitation for abstract words has been ascribed to a higher number of lexical associations^9^ and enriching emotional content (measured in terms of higher valence and arousal) for abstract than concrete words^8,11–13^. As a result, the original hypothesis of representational richness postulated by the DCT has been expanded beyond the distinction of verbal vs. sensorimotor information. Recent theoretical approaches highlight the importance of emotional information like valence and/or arousal for the conceptual representation and processing of abstract words (e.g., the *affective embodiment account*; for a review see^14^). According to rating studies, abstract concepts also comprise relatively more introspective, moral, social, temporal, spatial and magnitude information than concrete concepts^15–17^. The *representational substrates hypothesis*^4^ integrates those empirical findings and assumes a differential representational enrichment of concrete concepts by multimodal sensorimotor information and of abstract concepts by not only lexical but also emotional, social and magnitude-based information.

Further, research traced back abstract words’ lower context availability to their semantic diversity, i.e., their more variable meaning depending on the more diverse (and thus less easily retrievable) contexts they appear in compared to concrete words. In line with this, abstract words have been shown to pose higher demands on *semantic control* mechanisms, i.e., executive regulation processes, to successfully select and process the required word meaning^18,19^. Besides this quantitative difference in available contextual information and demands on semantic control processes, the *differential frameworks hypothesis*^20^ assumes a qualitative difference in how concrete and abstract words are organized in semantic memory. Rooted in how we experience concrete and abstract concepts’ referents in the real world as well as in linguistic contexts^4^, concrete words are thought to have taxonomic, perceptual similarity-based relations (e.g., butterfly and moth), while abstract words are rather related via thematic or contextual associations (e.g., wisdom and age). This qualitative difference takes effect as soon as concrete and abstract words are embedded in a similarity-based or associative context, which selectively facilitates concrete and abstract word processing^21,22^.

Current semantic processing theories integrate representational richness and semantic control as two distinct yet interacting processes^23,24^. Insights from electroencephalography (EEG) and functional magnetic resonance imaging (fMRI) suggested that representational richness and semantic control rely on distinct neural resources, involving the anterior temporal and the inferior frontal cortex, respectively^25–27^. In order to investigate behavioral correlates of semantic control and representational richness, Lambon Ralph et al. developed a synonym judgment task (SJT) and applied it in a series of studies with brain damaged patients. In the SJT, subjects choose a synonym to a concrete or abstract probe word from three test words, which requires deep semantic processing. Higher error rates for processing concrete vs. abstract words in semantic dementia patients (vs. healthy controls) indicated a causal involvement of anterior temporal regions in the processing advantage driven by representational richness^28–30^. Moreover, higher error rates for processing abstract vs. concrete words in aphasic patients with multimodal impairments of semantic comprehension after stroke-induced lesions in inferior frontal regions (including the ventrolateral prefrontal cortex) provided evidence for the causal involvement of such regions in semantic control demanded by abstract words^31,32^. While analyses focused on accuracy/error rates in patients, reaction times in the SJT turned out to be more sensitive to investigate semantic processing performance in healthy subjects^26,33,34^.

Only one study in this line of research investigated the interaction of representational substrates and semantic control by embedding the SJT in an elegant cueing paradigm^26^. In this paradigm, contextual and irrelevant cues, which consisted of two short sentences, preceded the concrete and abstract probe words. Contextual cues, which ended with the respective probe word, were thought to reduce demands on semantic control mechanisms (a previous study used a preliminary version of this paradigm^31^). By combining this paradigm with fMRI, Hoffman et al.^26^ confirmed that in the healthy brain, representational richness and semantic control mechanisms indeed recruit the previously identified distinct temporal and frontal brain areas, respectively. They further showed that the two mechanisms interact in their effect on behavioral correlates of semantic processing: Reaction times and error rates revealed not only a processing advantage for concrete over abstract words (i.e., the well-known CE) and after contextual over irrelevant cues (i.e., a contextual semantic facilitation effect), but crucially, that abstract word processing profited more strongly from contextual cues than concrete word processing.

The present study includes two experiments in order to replicate and further validate the finding of an interaction of concreteness and contextual relevance on reaction times in healthy adults by applying the SJT cueing paradigm^26^, which seems to be a powerful tool to investigate semantic processing comprehensively. In Experiment 1, we translated the original stimulus pool from English to German for a direct cross-language replication. We collected ratings on the translated stimuli for important psycholinguistic variables (i.e., imageability, context availability, arousal, valence). In Experiment 2, we aimed to further validate the SJT cueing paradigm by ruling out a potential alternative explanation to the original findings. Specifically, the repetition of the probe word at the end of the contextual cue in the original paradigm^26^ could not exclude the alternative explanation of the findings in terms of a mere lexical repetition rather than semantic facilitation effect. Lexical repetition vs. semantic facilitation effects result from different cognitive mechanisms^35–37^ and rely on distinct neural processes^38^. We thus reformulated the contextual cues in order to avoid the probe word repetition, thereby avoiding lexical repetition-priming (Experiment 2). In both Experiments, we expected to replicate the behavioral findings of Hoffman et al.^26^, i.e., a facilitation of word processing driven by word concreteness (main effect of concreteness) and contextual cues (main effect of cue), as well as a relatively stronger contextual facilitation for abstract words (concreteness by cue interaction effect).

## Experiment 1

### Method

#### Participants

Sixty-one healthy subjects voluntarily participated in Experiment 1. Based on the data obtained in the SJT, we excluded four participants whose percentage accuracy was more than 2.5 *SD* below the sample’s mean in at least one experimental condition and two participants whose mean reaction times were more than 2.5 *SD* above the sample’s mean in at least one experimental condition. The final sample consisted of 55 (38 females) German native speakers, between 18 and 33 years of age (*M* = 21.6 years, *SD* = 3.3 years). All reported to be right handed, which was confirmed by the Edinburgh Handedness Inventory^39^ ([EHI]; three participants scored between 0.37 and 0.47, which is considered bimanual, all others scored between 0.5 and 1, *M* = 0.80, *SD* = 0.19). All participants gave their written, informed consent and the study was approved by the ethics committee of the Faculty of Mathematics and Natural Sciences at Heinrich Heine University Düsseldorf and in line with the standards defined by the declaration of Helsinki.

#### Stimuli and material

Two independent translators translated the original stimuli by Hoffman et al.^26^ from English to German and an additional editor finalized the German stimuli. The primary goal was to keep the meaning as close as possible to the original stimuli. The stimuli consisted of 100 concrete and 100 abstract probe words, which could be nouns, verbs or adjectives. Additionally, there were three test words for each probe word: one semantically related target word and two unrelated foils. Finally, for each probe word there was a contextual and an irrelevant cue, which consisted of two short sentences. The contextual cue created a meaningful context for the probe and its second sentence ended with the probe word (see Table 1 for example stimuli). To create the irrelevant cue, we applied the same procedure as Hoffman et al.^26^: First, the 200 stimuli were divided into two sets, containing 50 concrete and 50 abstract probes each. Then, the contextual cues were randomly reassigned to the other probes within the same set. Half of the participants saw the first set with contextual and the second set with irrelevant cues, and vice versa. All words and sentences were presented in white letters on black background, in the font Arial, size 30 pt.

**Table 1:** Example stimuli used in Experiment 1 (A) and Experiment 2 (B).

		Cue sentences	Probe	Choices	
				Target	Foils
**A. Experiment 1**					
Concrete	cont	Es war ein sonniger Tag. Die Blumen lockten einen Schmetterling	Schmetterling *Butterfly*	Motte *Moth*	Vulkan, Sporthalle *volcano, gym*
		*It was a sunny day. The flowers attracted a butterfly*			
	irrel	Mein neues Kleid ist grün. Es ist mein liebstes Kleidungsstück			
		*My new dress is green. It is a beautiful garment*			
Abstract	cont	Ich bin mit Rassisten nicht einverstanden. Ich glaube an Toleranz	Toleranz *Tolerance*	Verständnis *Understanding*	Ironie, Bereich *irony, realm*
		*I disagree with racist people. I believe in tolerance*			
	irrel	Affen werden immer schlauer. Sie hören nicht auf, sich zu entwickeln			
		*Monkeys are getting smarter. They continue to evolve*			
**B. Experiment 2**					
Concrete	cont	Es war ein sonniger Tag. Draußen flatterten viele Insekten umher	Schmetterling *Butterfly*	Motte *Moth*	Vulkan, Sporthalle *volcano, gym*
		*It was a sunny day. Many insects fluttered around outside*			
	irrel	Ich mache Überstunden. Ich muss mir etwas dazuverdienen			
		*I am working late. I have to earn a bit extra*			
Abstract	cont	Ich bin gegen Rassismus. Ich versuche, vorurteilsfrei zu sein	Toleranz *Tolerance*	Verständnis *Understanding*	Ironie, Bereich *irony, realm*
		*I am against racism. I try to be open-minded*			
	irrel	Ich könnte mich dabei verletzen. Ich probiere es trotzdem			
		*I could hurt myself. I am trying it anyways*			

Example stimuli (original in German, with *English translation*) for one concrete and one abstract probe in the contextual (cont) and irrelevant (irrel) cue conditions.

#### Psycholinguistic variables

Table 2 displays the descriptive and inferential statistics on the psycholinguistic variables for concrete and abstract probes and their contextual cue sentences used in Experiment 1. The translation of the stimuli to German led to a higher mean length (number of letters) of abstract probes as well as sentences, both *p* ≤ 0.011. The word count of the sentences did not differ significantly, *p* = 0.145. Concrete and abstract probes did neither differ in their (written) frequency of occurrence in the corpus of the CELEX online database^40^, *p* = 0.951, nor in their (spoken) frequency of occurrence in the corpus of the SUBTLEX-DE database^41^, *p* = 0.525. We conducted online ratings of the probes on 7-point Likert scales of imageability, context availability, valence and arousal with 25 German native speakers (3 male), between 19 and 38 years of age (*M* = 23.9 years, *SD* = 5.9 years), who did not participate in the SJT experiment. Valence and arousal ratings were collected, as emotional content has been shown to have a strong influence on semantic processing of abstract words^8,11,12^. Additionally, we acquired ratings on the same scales for the cue sentences. This rating study was conducted post-hoc including the cues from Experiment 1 and 2, rated by 42 German native speakers (4 male), aged between 18 and 30 years (*M* = 20.7 years, *SD* = 2.6 years). Concrete words and sentences received higher mean ratings of imageability and context availability, as predicted by the DCT and the CAM, all *p* < 0.001. Concrete and abstract probes’ mean valence ratings did not differ significantly, *p* = 0.535, but concrete probes received a higher mean arousal rating, *p* = 0.017. Abstract cues received higher arousal and higher (negative) valence ratings than concrete cues, both *p* ≤ 0.042.

**Table 2:** Descriptive and inferential statistics of the psycholinguistic variables for the probe words (A) and cue sentences (B) used in Experiment 1*.*

Variable	Concreteness	Descriptive statistics		Inferential statistics		
		*M*	*SE*	*t*	*df*	*p*
**A. Probes**						
Length (letters)	Abstract	8.22	0.26	2.57	198	0.011
	Concrete	7.30	0.25			
Frequency (written)^a^	Abstract	50.96	8.72	0.06	185	0.951
	Concrete	50.08	11.18			
Frequency (spoken)^b^	Abstract	31.40	13.56	0.64	194	0.525
	Concrete	42.11	10.01			
Imageability	Abstract	2.64	0.11	20.54	198	< 0.001
	Concrete	5.83	0.11			
Context availability	Abstract	3.93	0.09	13.56	198	< 0.001
	Concrete	5.55	0.08			
Arousal	Abstract	2.78	0.11	2.41	198	0.017
	Concrete	3.17	0.12			
Valence	Abstract	0.20	0.12	0.62	198	0.535
	Concrete	0.31	0.13			
**B. Sentences**						
Length (letters)	Abstract	61.91	0.97	2.27	198	0.024
	Concrete	58.94	0.88			
Length (words)	Abstract	10.09	0.16	1.47	198	0.145
	Concrete	9.78	0.14			
Imageability	Abstract	3.38	0.10	12.33	187.59	< 0.001
	Concrete	4.92	0.08			
Context availability	Abstract	3.85	0.10	11.75	188.40	< 0.001
	Concrete	5.36	0.08			
Arousal	Abstract	3.19	0.11	2.05	198	0.042
	Concrete	2.86	0.12			
Valence	Abstract	− 0.34	0.12	2.21	198	0.024
	Concrete	0.03	0.12			

Imageability, Context Availability and Arousal were rated on 1–7 Likert scales, Valence on a scale from − 3 to + 3. Independent samples *t*-tests compared the psycholinguistic variables for concrete and abstract words. *n* = 100 per condition, except for frequency (written: *n*_concrete_ = 95, *n*_abstract_ = 92; spoken: *n*_concrete_ = 99, *n*_abstract_ = 97).

*M* mean, *SE* standard error, *df* degrees of freedom.

^a^Frequency of occurrence of Mannheim Lemmas in 1 Mio. words, based on the CELEX database^40^.

^b^Frequency of occurrence of case-insensitive lemmas based on the SUBTLEX-DE database^41^.

#### Procedure

**Figure 1:**
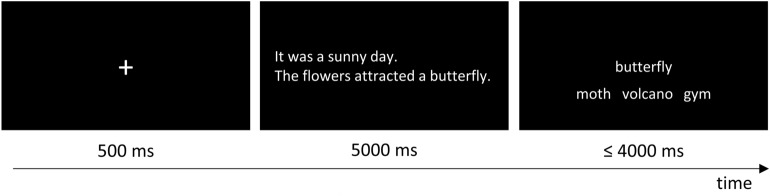
Timing and exemplary stimuli of one trial of the synonym judgment task with a contextual cue and a concrete probe.

We acquired the data in single subject testing sessions in two laboratory rooms at Heinrich Heine University. After providing detailed information about the experiment’s procedure, participants received standardized instructions over the computer monitor. Instructions included one contextual and one irrelevant cue example trial, which did not occur in the actual experiment. The experimenter read the instructions aloud and answered any arising questions in order to ensure effective comprehension of the task. The task was an SJT used previously in studies on patients and healthy subjects^26,28,31^, embedded in a cueing paradigm with contextual and irrelevant cues^26^. As illustrated in Fig. 1, each trial started with a fixation cross for 500 ms, followed by the cue for 5000 ms. Then, the probe appeared in the center of the screen and underneath it the three test words next to each other. The probe and test words were displayed on the screen till the participants’ response (see below) or for a maximum of 4000 ms. Three response buttons marked on a response box represented the test words’ position on screen. Throughout the experiment, the positions of the target word and the two foils were counter-balanced across the three possible positions and randomized over all the trials. Participants had to keep the digit, middle and ring finger of their right hand on the marked buttons of the response-box in order to reduce variability in reaction times due to hand posture. Participants were instructed to choose the test word, which was most similar to the probe, as fast and as accurately as possible via button press. Participants were informed that two short sentences, which might or might not end with the following probe word, would precede the presentation of the probe and test words and were asked to read those sentences thoroughly. Every 20 trials, there was the possibility to take a self-paced break. The experiment took approximately 35 min. After completion, participants received monetary compensation or course credit.

The software Presentation (version 17.2, www.neurobs.com) controlled the stimulus presentation and recorded the participants’ responses on a Windows 10 DELL PC. We used a 24′′ Asus LCD HDMI Monitor with a 1920 × 1080 Pixel resolution and a 60 Hz refresh rate. Participants gave their responses using three buttons of an RB-740 response box (Cedrus Corporation, San Pedro, USA).

#### Design and analysis

We measured reaction times starting from the presentation of the probe and test words until the participant’s response. Choices of the target word were considered correct, choices of the foils as errors. From all analyses, we excluded trials, in which the reaction time deviated more than 2.5 *SD* from the individual mean reaction time (resulting in *M* = 48.3 trials, *SD* = 1.7 trials with a minimum of 39 trials per participant per experimental condition: concrete-contextual, concrete-irrelevant, abstract-contextual, and abstract-irrelevant, total = 10,616 data points for the accuracy analysis). For the reaction time analysis, we also excluded trials with erroneous responses (resulting in *M* = 46.3 trials, *SD* = 2.9 trials with a minimum of 36 trials per participant per experimental condition, total = 10,194 data points).

We conducted linear mixed effects (LME) and generalized linear mixed effects (GLME) analyses, as they allow to control for interindividual variance introduced by participants and items^42^. For the sake of comparability with the original investigation^26^, we report the full results of factorial ANOVAs in the supplementary material (see Online Appendix A). For the (G)LME analysis, we used the lme4 package (version 1.1-21^43^) implemented in the R environment (version 3.6.3). Single-trial reaction times were entered into an LME analysis. We defined a model including the categorical fixed-effects factors Concreteness (concrete [+ 1], abstract [− 1]) and Cue (contextual [+ 1], irrelevant [− 1]) as well as their interaction as predictors for the reaction time data. We included Participants and Items as random-effect factors. For the Participants random-effect factor, we evaluated a linear model formula of Concreteness and Cue. We applied the restricted maximum likelihood approach to the LME analyses^44^ and used the Satterthwaite method to estimate degrees of freedom and significance for the model estimates, as implemented in the lmerTest package^45^ (version 3.1-1). Significant interactions were resolved via simple slope LME analyses as implemented in the R package jtool (version 2.0.3).

We analyzed binary accuracy data with a GLME analysis suitable for binomial distributions as implemented in the afex package^46^ (version 0.27-2). A model was defined that included the same fixed- and random effects factors defined above for reaction times. We applied the maximum likelihood approach to the GLME analysis (for validation of this approach see again Luke^44^). The likelihood-ratio test method was used based on type III sums of squares to estimate degrees of freedom and significance for the model estimates, as implemented in the glmer function included in the lme4 package. Additional (G)LME analyses including psycholinguistic variables as covariates were conducted to test for their potential confounding effect (see Online Appendix B) and including imageability as continuous predictor (see Online Appendix C).

### Results and discussion

**Figure 2:**
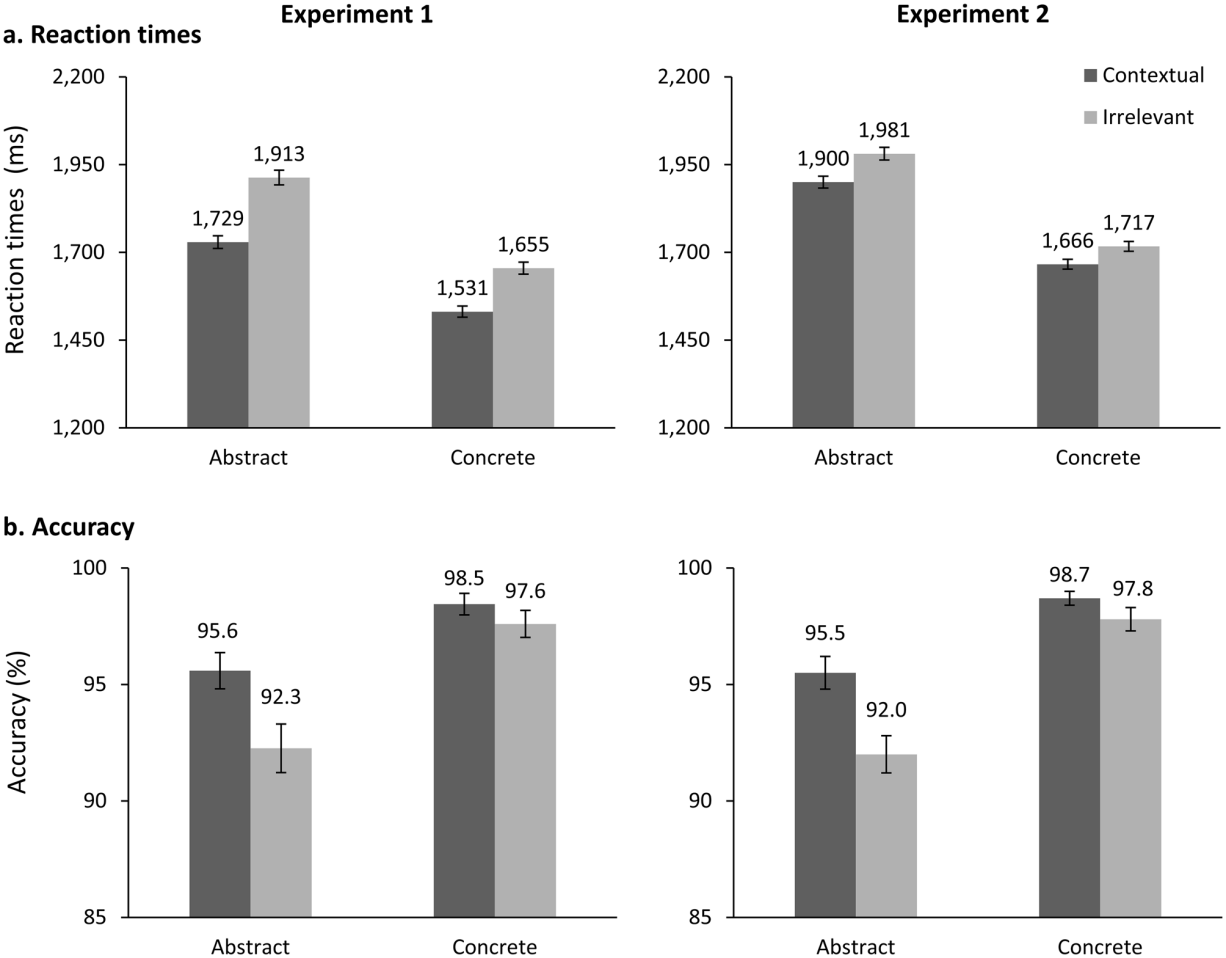
Descriptive statistics on mean reaction times (ms; (**a**)) and accuracy (%; (**b**)) in the synonym judgment task after contextual and irrelevant cues in Experiment 1 and Experiment 2. Error bars represent ± 1 standard error.

The (G)LME analyses revealed that Cue and Concreteness factors had significant main effects on the reaction time and accuracy data, all *p* < 0.001 (for descriptive statistics, see Fig. 2; for β estimates and effect-specific χ^2^/*t*-tests, see Table 3). The main effects were in line with our hypotheses, with faster and more accurate processing for concrete vs. abstract words (representing the CE) and contextual vs. irrelevant cues (representing the contextual semantic facilitation effect). For reaction times, the Concreteness × Cue interaction was significant, *p* < 0.001. We resolved the interaction via simple slope analyses with Cue as predictor and Concreteness as moderator and vice versa, to fully explore the interaction pattern. When Concreteness was the predictor and Cue was the moderator variable, we found a descriptively larger β estimate of the CE in the irrelevant than the contextual Cue condition, although both CEs were highly significant, *p* < 0.001. When Cue was the predictor and Concreteness was the moderator variable, we found that the contextual semantic facilitation effect on reaction times had a descriptively smaller β estimate for concrete than abstract words, although both *p* < 0.001. This interaction pattern on reaction times was in line with our hypotheses: Abstract word processing profited relatively more strongly from a relevant cue than concrete word processing, although concrete words were processed faster (i.e., showed a CE) also in the contextual cue condition. For accuracy, the Concreteness × Cue interaction was not significant, *p* = 0.586. Descriptively, the accuracy result pattern was in line with our hypothesis and mirrored the reaction times: relevant cues facilitated abstract as well as concrete word processing, though with a stronger impact on abstract word processing. Also previous studies found reaction times to be more sensitive to modulations of semantic processing performance than accuracy in the SJT in healthy subjects^26,33,34^. Possibly, a ceiling effect (all conditions > 90% accuracy), especially for concrete words restricted the possible range of context effects and might have kept the interaction effect from reaching significance (see Jaeger^47^ for a detailed discussion). A more challenging task (e.g., by implementing target words, which are only synonyms to the probes in the given context; by using stronger distractors or by shifting the speed-accuracy tradeoff towards faster and thus more error-prone responses) might have led to a significant interaction in healthy controls also for the accuracy. Notably, the factorial ANOVA yielded a significant interaction effect (see Appendix A in the Supplementary Material). Taken together, the missing interaction effect on accuracy in the GLME accuracy analysis does not challenge the original results or the validity of the paradigm. Instead, it highlights that semantic processing speed, rather than accuracy, reflects effects of semantic richness and contextual facilitation in healthy adults.

**Table 3:** Βeta estimates and their standard error, estimated degrees of freedom, t-/χ^2^ and *p*-values for (G)LME analyses with the factors Concreteness and Cue on single-trial reaction times (A) and accuracy (B) in the synonym judgment task in Experiment 1 and 2.

Effect	Experiment 1					Experiment 2				
	β Estimate	*SE*	*df*	*t*	*p*	β Estimate	*SE*	*df*	*t*	*p*
**A. Reaction times (ms)**										
Concreteness	− 118.11	14.74	207.25	− 8.02	< 0.001	− 133.73	16.56	217.92	− 8.08	< 0.001
Cue	− 79.58	4.59	53.60	− 17.34	< 0.001	− 36.94	3.69	81.03	− 10.02	< 0.001
Concreteness × Cue	15.45	3.65	9845.54	4.23	< 0.001	8.36	3.13	14,780.15	2.67	0.008
Simple slope analyses										
Concreteness as predictor										
Contextual Cues	− 102.66	15.15		− 6.78	< 0.001	− 125.37	16.84		− 7.45	< 0.001
Irrelevant Cues	− 133.56	15.21		− 8.78	< 0.001	− 142.09	16.87		− 8.42	< 0.001
Cue as predictor										
Concrete probes	− 64.13	5.78		− 11.10	< 0.001	− 28.59	4.73		− 6.05	< 0.001
Abstract probes	− 95.02	5.95		− 15.97	< 0.001	− 45.24	4.55		− 9.95	< 0.001

Effect	β Estimate	*SE*	χ^2^	*df*	*p*	β Estimate	*SE*	χ^2^	*df*	*p*
**B. Accuracy (%)**										
Concreteness	0.58	0.11	27.62	1	< 0.001	0.72	0.11	44.13	1	< 0.001
Cue	0.29	0.06	18.36	1	< 0.001	0.30	0.05	24.99	1	< 0.001
Concreteness × Cue	− 0.03	0.06	0.30	1	0.586	− 0.02	0.04	0.23	1	0.631

Simple slope analyses with Concreteness as predictor investigated the effect of Concreteness within the relevant/irrelevant cue condition. Simple slope analyses with Cue as predictor investigated the effect of Cue within the concrete/abstract condition.

*SE* standard error, *df* degrees of freedom.

Experiment 1 thus replicated the behavioral findings of the original study with English stimulus material^26^ in the German language, while controlling for random effects introduced by participants as well as items. Notably, the contextual cue in Experiment 1 as well as in the original study by Hoffman et al.^26^ ended with the probe word, which thus appeared repeatedly on screen. A processing facilitation by mere lexical repetition rather than semantic information (i.e., semantic priming) is thus an alternative explanation for the behavioral results of Experiment 1 and Hoffman et al.^26^. Lexical repetition and semantic priming have not only been shown to have additive effects on reaction times^35^ and rely on distinct cognitive mechanisms^36^, but also to rely on distinct neural substrates^38^, partially overlapping with the regions identified to be correlates of semantic control processes in the fMRI study by Hoffman et al.^26^. In Experiment 2, we thus reformulated the contextual cue avoiding the probe word to test whether contextual information without repetition of the probe word would lead to comparable results. A successful replication of the findings from Experiment 1 after this modification would strengthen the interpretation of the findings in terms of truly semantic context effects rather than repetition effects. Furthermore, in the translated stimulus pool in Experiment 1, abstract and concrete probe words differed significantly regarding their length (i.e., number of letters) and probe words and cue sentences differed regarding arousal ratings. Although in Experiment 1 we controlled for item-introduced variability by including Items as a random effects factor in the LME models, in Experiment 2 we carefully matched concrete and abstract probes for word length and probe words and cue sentences for their arousal ratings.

## Experiment 2

### Method

#### Participants

Eighty-six subjects voluntarily participated in Experiment 2. Based on the data from the SJT, we excluded three participants whose percentage accuracy was more than 2.5 *SD* below the sample’s mean in at least one experimental condition. The final sample consisted of 83 (59 females) healthy German native speakers, between 18 and 37 years of age (*M* = 22.0 years, *SD* = 3.7 years). All reported to be right-handed (EHI of eight participants scored between 0.24 and 0.47, which is considered bimanual, all others scored between 0.5 and 1, *M* = 0.80, *SD* = 0.20). All participants gave their written, informed consent and the study was approved by the ethics committee of the Faculty of Mathematics and Natural Sciences at Heinrich Heine University Düsseldorf and was in line with the standards defined by the declaration of Helsinki.

#### Stimuli and material

For Experiment 2, we changed some of the translated stimuli from Experiment 1. Because in the original study adjectives occurred as probe words exclusively for the abstract category, we replaced the abstract adjectives by the corresponding abstract verbs or nouns in order to reduce variability of the grammatical class across the two conditions. In order to match concrete and abstract probes for letter length, we replaced some of the probes by semantically closely related words with a different length (number of letters). Most importantly, we reformulated the contextual cues so that they did not end with the probe word. Whenever possible, we left the first cue sentence unchanged. After reformulation, the sentences contained neither the probe word, nor a direct synonym, nor antonym, nor a word derived from the same root. Furthermore, the last word of the second sentence was always from a different grammatical class than the probe in order to avoid that the probe word could be integrated into the sentential context at the morpho-syntactic level. This should assure that any facilitative context effects should arise only at the semantic level. We also replaced all negations with positive formulations. The two cue sentences mostly described a situation, in which the probe word could occur. Some paraphrased or gave a definition of the probe’s meaning (see Table 1 for example stimuli).

#### Psycholinguistic variables

Table 4 displays the descriptive and inferential statistics on the psycholinguistic variables for concrete and abstract probes and their contextual cue sentences in Experiment 2. Concrete and abstract probes and sentences did not differ in their length (number of letters or words), all *p* ≥ 0.324. Concrete and abstract probes did neither differ in their (written) frequency nor in their (spoken) frequency of occurrence, *p* = 0.164. As in Experiment 1, we acquired ratings on imageability, context availability, valence and arousal, and additionally concreteness and association with emotional experience (for instructions see Appendix D in the Supplementary Material). The rating sample consisted of 29 (5 male) participants, aged between 18 and 42 years (*M* = 22.9 years, *SD* = 5.7 years). We further collected the same ratings for the cues as in Experiment 1 (see above for sample details). All participants in the ratings were German native speakers and none of them participated in the SJT experiment. In the pool of stimuli for Experiment 2, the abstract and concrete probes and sentences were matched for length (number of letters, number of words) and the probes were matched for their frequency of occurrence, all *p* ≥ 0.324. Concrete probe words received higher ratings on the scales of imageability, context availability and concreteness, all *p* < 0.001. Concrete sentences received higher context availability, *p* < 0.001, but not imageability ratings, *p* = 0.293. Concrete and abstract probes and sentences did not differ significantly in their mean ratings on the scales of valence, arousal and association with emotional experience, all *p* ≥ 0.098. In order to gain quantitative and qualitative information regarding the relation of the probes and their target words, we conducted additional ratings on a similarity (i.e., “How similar are the two words? How well can you put them in a common category?”) and association strength scale (i.e., “How strongly associated are the two words? How well do they form a common context?”) motivated by the assumptions of differential representational frameworks of concrete and abstract words^20^. The probe-target pairs were rated on these scales by 25 (3 male) German native speakers, aged between 19 and 46 years (*M* = 24.4 years, *SD* = 7.2 years). Concrete words received higher ratings on both scales, *p* ≤ 0.033 (see Table 4C).

**Table 4:** Descriptive and inferential statistics of the psycholinguistic variables for the probe words (A), cue sentences (B) and relation strength between probe and target words (C) used in Experiment 2.

Variable	Concreteness	Descriptive statistics		Inferential statistics		
		*M*	*SE*	*t*	*df*	*p*
**A. Probes**						
Length (letters)	Concrete	7.71	0.25	1.00	198	0.324
	Abstract	8.05	0.24			
Frequency (written)^a^	Concrete	47.66	11.13	0.02	186	0.981
	Abstract	47.99	8.28			
Frequency (spoken)^b^	Concrete	38.48	9.69	1.40	194	0.164
	Abstract	22.17	6.48			
Imageability	Concrete	6.06	0.06	31.37	198	< 0.001
	Abstract	3.00	0.79			
Context availability	Concrete	5.74	0.04	17.03	153.25	< 0.001
	Abstract	4.29	0.07			
Concreteness	Concrete	6.16	0.08	34.65	198	< 0.001
	Abstract	2.54	0.07			
Arousal	Concrete	2.53	0.10	0.52	198	0.605
	Abstract	2.60	0.09			
Valence	Concrete	0.36	0.11	0.58	198	0.562
	Abstract	0.28	0.10			
Emotional experience	Concrete	2.94	0.14	1.66	198	0.098
	Abstract	2.62	0.14			
**B. Sentences**						
Length (letters)	Concrete	58.67	0.89	0.44	190.81	0.663
	Abstract	59.28	1.08			
Length (words)	Concrete	9.72	0.17	0.66	198	0.512
	Abstract	9.88	0.18			
Imageability	Concrete	4.36	0.15	1.05	198	0.293
	Abstract	4.12	0.17			
Context availability	Concrete	5.08	0.09	6.65	186.96	< 0.001
	Abstract	4.14	0.11			
Arousal	Concrete	2.50	0.13	0.387	198	0.699
	Abstract	2.44	0.12			
Valence	Concrete	4.05	0.10	1.43	198	0.154
	Abstract	3.85	0.10			
**C. Probe-target relation strength**						
Similarity	Concrete	5.82	0.06	2.15	198	0.033
	Abstract	5.63	0.06			
Association	Concrete	5.50	0.07	2.99	198	0.003
	Abstract	5.20	0.07			

Imageability, Context Availability, Concreteness, Emotional Experience, Arousal as well as the strength of the probe-target relation based on Similarity and Association were rated on 1–7 Likert scales, Valence on a scale from − 3 to + 3. Independent samples *t*-tests compared the psycholinguistic variables for concrete and abstract words. *n* = 100 per condition, except for frequency (written: *n*_concrete_ = 96, *n*_abstract_ = 92; spoken: *n*_concrete_ = 100, *n*_abstract_ = 96).

*M* mean, *SE* standard error, *df* degrees of freedom.

^a^Frequency of occurrence of Mannheim Lemmas in 1 Mio. words, based on the CELEX database^40^.

^b^Frequency of occurrence of case-insensitive lemmas based on the SUBTLEX-DE database^41^.

#### Procedure

We applied the same experimental procedure as in Experiment 1.

#### Design and analysis

The same design and data analysis as in Experiment 1 were applied. On average, 47.7 trials (*SD* = 2.2 trials, with a minimum of 40 trials) per participant per condition were entered into the accuracy analysis (total = 15,878 data points). On average, 45.8 trials (*SD* = 3.5 trials, with a minimum of 32 trials) per participant per condition were entered into the single-trial reaction time analysis (total = 15,204 data points). We conducted additional (G)LME analyses including psycholinguistic variables as covariates to test for their potential confounding effect (see Online Appendix B) and including imageability as continuous predictor (see Online Appendix C).

### Results and discussion

Also in Experiment 2, the (G)LME analyses revealed that the factors Cue and Concreteness had significant effects on the reaction time as well as accuracy data, all *p* ≤ 0.001 (for descriptive statistics, see Fig. 2; for β estimates and effect-specific χ^2^/*t*-tests, see Table 3). Again, the effects were in line with our hypotheses, with faster and more accurate processing for concrete vs. abstract words and for contextual vs. irrelevant cues. For reaction times, the interaction of Cue and Concreteness was significant, *p* = 0.008. We resolved this interaction as described above in Experiment 1. When Cue was the moderator variable, we found a descriptively larger CE in the irrelevant than the contextual Cue condition, although both CEs were highly significant, *p* < 0.001. When Concreteness was the moderator variable, the contextual semantic facilitation effect on reaction times was descriptively smaller for concrete than abstract words, although it was significant in both conditions, *p* < 0.001. The pattern of the interaction was again in line with our hypotheses and reflected that abstract words profited more strongly from a contextual cue, with a CE still present in the contextual cue condition. As in Experiment 1, the interaction of Cue and Concreteness on accuracy did not reach significance *p* = 0.924 but mirrored the reaction time pattern descriptively (as for Experiment 1, the factorial ANOVA yielded a significant interaction, see Appendix A in the supplementary material). In summary, after we eliminated potential confounds by repetition-priming and uncontrolled differences in emotional psycholinguistic variables in Experiment 2, we still replicated the result pattern found in the original study^26^.

## General discussion

The results from Experiment 1 and 2 replicated the behavioral findings of concreteness and context effects on word processing in a synonym judgment task with healthy subjects^26,31^. For reaction times, we also replicated the interaction of concreteness and context effects, with abstract word processing profiting more strongly from a contextual cue. Ceiling effects in the accuracy data possibly kept the interaction effect from reaching significance^47^, however it mirrored the reaction time pattern on the descriptive level. The behavioral result pattern was thus highly consistent between the original study, its direct replication in Experiment 1 and the replication with the modified stimulus material in Experiment 2. Despite the moderate sample sizes, this successful replication delivers important validation of previous research^48,49^, especially as the literature yielded contradictive evidence regarding the CE^7–9,50^, and the interplay between semantic control and representational richness has only been investigated in few behavioral paradigms^7,26,31,50^.

Crucially, Experiment 2 further validated the findings of the original study and Experiment 1 as we eliminated the potential confounding influence of mere lexical repetition on the context effects. Given the comparable behavioral result patterns in Experiments 1 and 2, it seems unlikely that largely different cognitive processes were induced by the additional repetition priming in Experiment 1. It seems, however, that the repetition priming has been added to the purely semantic priming effects, as the highly significant main effect of the cue had a notably higher β estimate (see Table 3) and reaction times were lower in the contextual condition (see Fig. 2) in Experiment 1 than 2. There is, however, some evidence that repetition and semantic priming effects interact with concreteness in an at least partially distinct way. A qualitative comparison of the results obtained in Experiment 1 and 2 shows that also the Concreteness × Cue interaction effect had a higher β value in Experiment 1. Looking at Fig. 2, this might be due to a less pronounced difference in priming effects between concrete and abstract words. Thus, the additional repetition priming in Experiment 1 might have favored abstract word processing especially, possibly due to their more context-dependent meanings^4^. However, whether repetition priming vs. semantic priming in an SJT rely on comparable or at least partially distinct neural correlates^38^ and cognitive processes^36^ is beyond the scope of this purely behavioral study, especially as Experiment 1 did not investigate pure repetition priming but semantic priming contributed to the results of both experiments.

The resolution of the Concreteness × Cue interaction further revealed a reduced but still present CE in the contextual cue conditions in both experiments for reaction times and descriptively also for accuracy. This residual CE contradicts assumptions of the CAM, which postulates that contextual information erases CEs completely^2,6,51^. In line with our results, previous studies found a residual behavioral^7,26^ or electrophysiological CE in the presence of contextual cues^25^. However, our behavioral measures were not just sensitive to processes elicited by the probes’ psycholinguistic features, but also to target selection processes required by the SJT. The targets were designed to be synonyms and thus their relation to the probes was based more strongly on semantic similarity than association, which might have favored concrete word processing^21^. Taken together, our findings support the notion that representational richness and demands on semantic control mechanisms elicit distinct cognitive processes, which interact on the behavioral level. In the bigger picture, our results thus support theoretical approaches that integrate representational content and semantic control mechanisms in order to explain semantic word processing comprehensively^4,23–25^.

When drawing conclusions regarding the role of certain psycholinguistic variables, it is important to acknowledge the multitude of often correlated variables affecting semantic processes^7,9^. The concreteness, imageability, and context availability ratings validated our manipulation of stimulus concreteness and confirmed the assumption that concrete words score higher on these scales^5,50^, reflecting their representational richness. We further controlled for a number of potential confounds by not only carefully measuring (and matching, in Experiment 2) the psycholinguistic variables of our probe words and cue sentences but also by including probe length, written and spoken frequency, emotional variables (valence, arousal, association with emotional experience) and the probe-target similarity and association strength separately as covariates into additional (G)LMEs reported in the supplementary material (see Online Appendix B). Importantly, these additional models validated our findings, as eliminating the influence of the psycholinguistic variables by including them as covariates did not change the inferential result pattern of the concreteness and cue effects reported above. For both experiments, the additional analyses showed that the emotional variables had a significant facilitative effect on word processing, without changing the inferential pattern of the concreteness and cue effects. However, controlling for affective psycholinguistic variables as we did by matching valence, arousal and emotional experience between abstract and concrete probe words in Experiment 2 might to some degree restrict the external validity of our findings, as previous research on semantic word processing pointed out that affective content is especially important for abstract concepts^8,11,52^, as highlighted in the affective embodiment account^14^. Instead of avoiding effects of emotional psycholinguistic variables, it might be fruitful to systematically investigate them in future research using the SJT cueing-paradigm.

An important limitation to the interpretability of our results is the assumption of a concrete-abstract dichotomy implied by the concreteness manipulation. Although previous research often investigated concreteness in this dichotomous manner^5,7,9,20,26^, some studies suggest that a continuous representation of concreteness is more suitable^15,22,53^. The interested reader can find an additional LME analysis on the reaction times including imageability as continuous predictor in the supplementary material (see Online Appendix C). This analysis mirrors the pattern found in the analyses with the factorial factor described above. However, a limitation of these additional analyses is that imageability ratings were not normally distributed, as the original stimulus selection aimed at a clear abstract-concrete dichotomy. Future research should use stimulus pools suitable to fully exploit the potential of LME reaction time analyses with parametric modulations of continuous variations of semantic features. Furthermore, although concreteness and imageability ratings of the probes used in Experiment 2 were highly correlated (*r* = 0.971, *p* > 0.001), it is important not to consider these variables as fully interchangeable. Previous research showed that concreteness and imageability can lead to at least partially dissociated electrophysiological processes^9,15,54^, even though they tap into the same mechanisms of enriching concrete word representations with sensory experience.

Another limitation is that reaction times in the SJT might reflect a conglomerate of cue-sentence, probe and test word-driven processes in an additive manner but these processes could also interact at different stages before behavioral output (for ERP evidence on early semantic processing stages see Hinojosa et al.^55^). A promising approach to address these limitations would be to combine this paradigm with electro- or magnetoencephalographic recordings, which could deliver important insights into the temporal dynamics of semantic processes involved^56,57^. Further, we relied on previous studies using the SJT cueing paradigm in our assumption that the participants thoroughly read the cue sentences, as requested in the instructions. Our paradigm, however, did not allow us to verify this assumption and future adaptation of the paradigm could include catch trials, in which participants answer questions about the cues.

In conclusion, this study corroborated the behavioral results of Hoffman et al.^26^ in two experiments with carefully controlled psycholinguistic variables by ruling out important alternative explanations of lexical repetition effects. Our findings deliver evidence that semantic processing differences driven by an interaction of representational substrates and semantic control mechanisms can be generalized over different languages and tasks^7,25,26^. Future research might focus on more fine-grained investigations of how a continuous manipulation of representational richness and demands on semantic control mechanisms affect semantic processing with stimuli designed especially for this purpose. Given the heterogeneous findings on CE in semantic processing, replications as the study at hand applying well-designed and controlled paradigms like the cueing-paradigm of Hoffman, et al.^26^ are crucial to gain a comprehensive understanding of the organization and principles of semantic knowledge.

## Supplementary Information

Supplementary Information.

## Data Availability

The datasets generated during and/or analyzed during the current study are available from the corresponding author on reasonable request.
