# Supplementary material for: Corroborating behavioral evidence for the interplay of representational richness and semantic control in semantic word processing
Source: Sci Rep. 2021 Mar 17;11:6184. doi: 10.1038/s41598-021-85711-7 (PMC7971068; doi:10.1038/s41598-021-85711-7)
Supplement: Supplementary file 1 — Supplementary Information. [file 41598_2021_85711_MOESM1_ESM.pdf]

Supplementary online material to**:**

Corroborating behavioral evidence for the interplay of representational richness and semantic control in semantic word processing

Laura Bechtold, Christian Bellebaum, Paul Hoffman, and Marta Ghio

Appendix A: Factorial ANOVAs

Design and Analysis

Statistical analyses were performed with IBM SPSS statistics (version 23). We analyzed the accuracy and reaction time in the SJT in dependence of the Concreteness (abstract, concrete) and Cue (irrelevant, contextual) via separate 2 × 2 repeated measures analyses of variance (ANOVAs). Interactions were resolved via dependent samples *t*-tests.

Results

In Experiment 1, we found significant main effects of Concreteness and Cue as well as a significant Concreteness × Cue interaction for both the percentage accuracy as well as the reaction time in the SJT, all $p < .001$. Dependent samples *t*-tests confirmed that both, abstract and concrete words showed a significant processing advantage in terms of more accurate and faster responses after a contextual cue than an irrelevant cue, all $p \leq .004$. Dependent samples *t*-tests comparing abstract and concrete word processing showed that the concreteness effect was present within the contextual as well as irrelevant cue condition for both measures, all $p < .001$. Further, the processing advantage after a contextual vs. irrelevant cue was significantly larger for abstract than concrete words for accuracy (mean difference = 2.8%, SE = 0.8%) as well as reaction time (mean difference = 71 ms, SE = 15 ms), both $p < .001$.

In Experiment 2, we found significant main effects of Concreteness and Cue on both accuracy and reaction times, all $p < .001$.We also found a significant Concreteness × Cue interaction for both the percentage accuracy, $p < .001$, as well as the reaction time, $p = .020$. The resolution of the interaction with dependent samples *t*-tests mirrored the findings of Experiment 1 as well. Abstract and concrete words showed a significant processing advantage after a contextual cue compared to an irrelevant cue, all $p \leq .002$. The concreteness effect was again significant within the contextual and irrelevant cue condition for both measures, all $p < .001$. The processing advantage after a contextual vs. irrelevant cue was significantly larger for abstract than concrete words for accuracy (mean difference = 2.5%, $SE = 0.6\%$), $p < .001$, as well as reaction time (mean difference = 29 ms, $SE = 12$ ms), $p = .020$, which is again in line with our hypotheses.

Appendix B: Additional covariate analysis

Analysis

We performed additional analyses to investigate the influence of potentially confounding psycholinguistic variables on the results reported in the main article. We did not include all the psycholinguistic variables as predictors into one model to avoid the problem of multicollinearity with correlated predictors (Pearson correlation coefficients are displayed in Table B1). Instead, we tested a series of models, each including one psycholinguistic variable as an additional continuous fixed-effect covariate into the (G)LMs specified in the main article. Specifically, for Experiments 1 and 2 we tested five models, each including Concreteness and Cue as categorical fixed-effects factors as well as their interaction, and Participants (with a linear model formula of Concreteness and Cue) and Items as random-effect factors. In addition, model(1) included the probe length (number of letters), model(2) the written word frequency, model(3) the spoken word frequency, model(4) the absolute valence (irrespective of polarity) and model(5) the arousal ratings as covariate. For Experiment 2 we additionally specified model(6) including the probes' association with emotional experience, model(7) including the probe-target similarity and model(8) including the probe-target association strength as covariate. For all the additional continuous fixed-effects covariates the values were mean centered.

Results

Crucially, the inferential pattern of the Cue and Concreteness main and interaction effects on reaction times and accuracy described in the main article did not change in any of the eight models including additional covariates. Specifically, for all models the (G)LME analyses revealed that Cue and Concreteness factors had significant main effects on the reaction time and accuracy data, all $p < .001$, while the Cue × Concreteness interaction was significant only for reaction time, all $p < .001$ (Experiment 1), all $p \leq .008$ (Experiment 2),

but not for accuracy, all $p \geq .574$. The consistent pattern of results across all the tested models, each including a psycholinguistic variable as covariate, validated our a-priori matching of psycholinguistic variables for abstract and concrete words.

We additionally report here the β estimates of the covariate' effect for each tested model and effect-specific χ^2/t-tests (see Table B2), and we provide an interpretation of such additionally findings.

Model(1). Probe length had a significant effect on reaction times but not accuracy. This might be due to a reduced reading speed, which previous research linked to word length in healthy adults, when words were longer than 5 letters [1], which applies to 166 of our 200 probe words and might thus have influenced reaction times.

Model(2 and 3). Neither written nor spoken word frequency had a significant effect as a covariate. Effects of word frequency have previously been shown for lexical decision times [2]. However, the processes involved in synonym judgments might rely on semantic rather than lexical variables to a greater extent [3], which might explain why written and spoken word frequency did not affect our results.

Model(4,5,6). The covariates valence and arousal had significant effects on reaction times as well as accuracy. Both emotional variables led to lower reaction times and higher accuracy. Processing facilitation by emotional information is in line with mechanisms of semantic enrichment assumed by the representational substrates hypothesis [4] as well as the affective embodiment account [5], with the latter approach assuming that such mechanisms take place only for abstract words. For Experiment 2, just like valence and arousal, also emotional experience benefitted semantic processing performance and showed a significant main effect on reaction times and accuracy.

Model(7 and 8). In Experiment 1, probe-target similarity and association strength as covariates had significant effects on reaction times as well as accuracy. Both variables led to

lower reaction times and higher accuracy. Higher values for both variables seem to have facilitated the correct identification of the synonym.

Table B1

Bivariate Pearson correlation coefficients (r) of the psycholinguistic variables involved in the additional analyses for Experiment 1 (a) and Experiment 2 (b).

a. Experiment 1

		Length	CELEX	SUBTLEX	Arousal
CELEX	*r*	-.233**			
	p	.001			
SUBTLEX	*r*	-.193**	.526**		
	p	.006	< .001		
Arousal	*r*	-.081	-.004	.091	
	p	.255	.953	.201	
Valence	*r*	-.011	-.025	.158*	.778**
	p	.877	.728	.025	< .001

b. Experiment 2

		Length	CELEX	SUBTLEX	Arousal	Valence	Emot. Exp.	Similarity
CELEX	*r*	-.251**						
	p	< .001						
SUBTLEX	*r*	-.268**	.551**					
	p	< .001	< .001					
Arousal	*r*	-.122	.093	.234**				
	p	.087	.191	.001				
Valence	*r*	-.038	.028	.229**	.736**			
	p	.590	.696	.001	< .001			
Emot. Exp.	*r*	-.074	.076	.265**	.890**	.821**		
	p	.302	.283	< .001	< .001	< .001		
Similarity	*r*	-.055	.076	.128	.188**	.215**	.195**	
	p	.443	.286	.071	.008	.002	.006	
Association	*r*	-.035	.037	.028	.131	.128	.134	.909**
	p	.621	.605	.695	.064	.071	.059	< .001

Note. * $p < .05$, ** $p < .01$, *** $p < .001$.

Table B2

Beta estimates and their standard error, estimated degrees of freedom, t-/ χ^2 and p-values for the covariates included additionally into the (G)LME analyses on single-trial reaction times (left) and accuracy (right) in Experiment 1 (a) and Experiment 2 (b).

**a. Experiment 1**	Reaction times					Accuracy				
	β Estimate	*SE*	*t*	*df*	*p*	β Estimate	*SE*	χ^2	*df*	*p*
(1)Length	22.73 ***	5.46	4.17	194.25	< .001	-0.05	0.04	1.49	1	.222
(2)Celex	-0.13	0.15	-0.85	192.84	.394	< 0.01	0.00	0.14	1	.712
(3)Subtlex	-0.14	0.12	-1.11	194.51	.268	< 0.01	0.00	0.02	1	.901
(4)Valence	-43.58 *	19.30	-2.26	193.24	.025	0.37 **	0.15	7.62	1	.006
(5)Arousal	-35.26 *	13.74	-2.57	192.89	.011	0.22 *	0.11	4.77	1	0.029

**b. Experiment 2**										
	β Estimate	*SE*	*t*	*df*	*p*	β Estimate	*SE*	χ^2	*df*	*p*
(1)Length	17.47 **	6.35	2.75	195.19	.007	-0.02	0.05	0.16	1	.690
(2)Celex	-0.14	0.17	-0.83	195.01	.409	0.00	0.00	0.05	1	.829
(3)Subtlex	-0.34	0.20	-1.72	194.42	.087	0.00	0.00	0.80	1	.370
(4)Valence	-81.59 **	24.15	-3.38	194.86	.001	0.33 *	0.17	4.37	1	.037
(5)Arousal	-70.55 ***	16.83	-4.19	194.59	< .001	0.26 *	0.12	5.33	1	.021
(6)Emo.Experience	-47.96 ***	12.70	-3.78	194.89	< .001	0.25 **	0.09	8.50	1	.004
(7)Similarity	-206.78 ***	17.51	-11.81	198.25	< .001	0.69 ***	0.14	25.91	1	< .001
(8)Association	-218.87 ***	22.23	-9.85	198.52	< .001	0.70 ***	0.17	19.21	1	< .001

Note. * $p < .05$, ** $p < .01$, *** $p < .001$.

Appendix C: Reaction time LME with Imageability as a continuous factor

Analysis

We entered reaction times in an LME analysis as specified in the main article with one modification: We included the mean centered imageability ratings as a continuous predictor instead of the factorial dichotomous factor concreteness. The model thus included Cue (relevant/irrelevant) and Imageability (continuous) as fixed effects as well as Participant (with a linear model formula for Cue and Imageability) and Item as random effects.

Results

In Experiments 1 and 2, both main effects and the interaction were highly significant (for descriptive statistics, see Figure C1 and C2, respectively; for β estimates and effect-specific χ^2/*t*-tests, see Table C1). Please note that Imageability was not evenly distributed, due to the (methodologically introduced) dichotomy of concrete and abstract stimuli.

Table C1

Beta estimates and their standard error, estimated degrees of freedom, t- and p-values for LME analyses with the factors Imageability and Cue on single-trial reaction times in Experiment 1 (left) and Experiment 2 (right).

	Experiment 1					Experiment 2				
Effect	β Estimate	*SE*	*df*	*t*	*p*	β Estimate	*SE*	*df*	*t*	*p*
Imageability	-68.71	7.66	211.00	-8.97	< .001	-89.21	9.27	222.19	-9.63	< .001
Cue	-79.10	4.61	53.53	-17.17	< .001	-36.56	3.70	80.70	-9.89	< .001
Imageability × Cue	7.33	1.95	9862.24	3.75	< .001	8.37	1.85	14787.63	4.53	< .001
Simple Slope Analyses										
Imageability as predictor										
Contextual Cues	-61.38	7.88		-7.79	< .001	-80.84	9.44		-8.56	< .001
Irrelevant Cues	-76.03	7.92		-9.60	< .001	-97.58	9.46		-10.32	< .001
Cue as predictor										
High Imageability	-65.39	5.87		-11.15	< .001	-22.43	4.83		-4.64	< .001
Low Imageability	-92.80	5.89		-15.76	< .001	-50.64	4.43		-11.44	< .001

Note. *SE* = standard error, *df* = degrees of freedom. Simple slope analyses with Imageability as predictor investigated the effect of Imageability within the contextual/irrelevant cue condition. Simple slope analyses with Cue as predictor investigated the effect of Cue within the high (+1 *SD*) and low (-1 *SD*) Imageability condition.

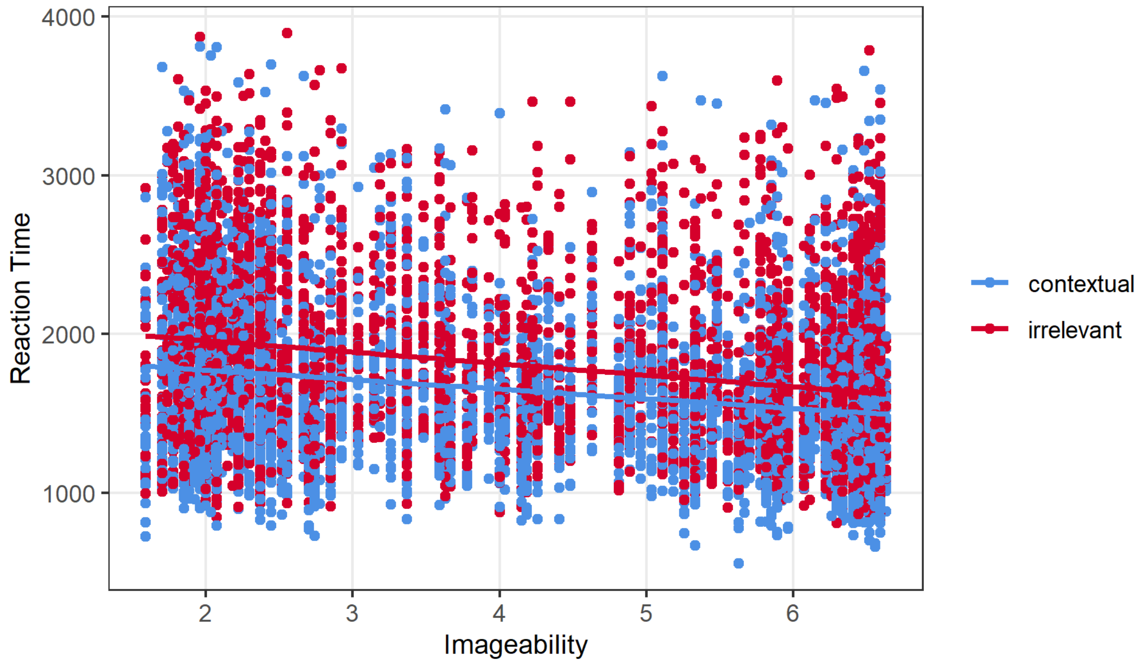

Figure C1. Reaction time (ms) in Experiment 1 depending on Imageability (continuous ratings on a 1-7 Likert scale) and Cue (contextual, irrelevant).

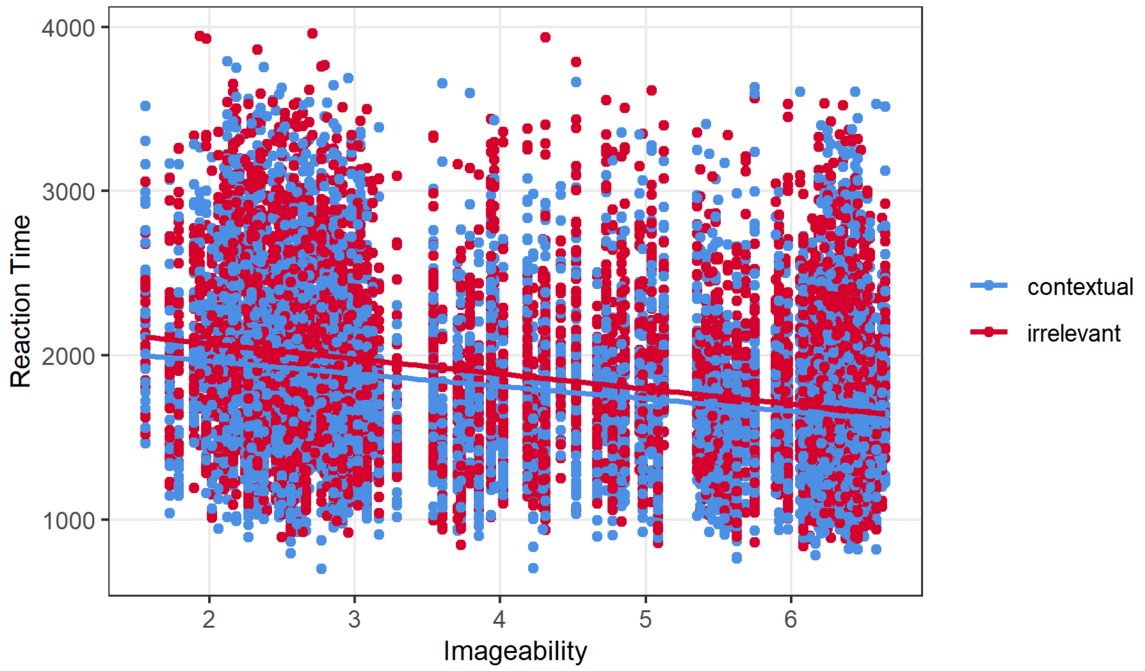

Figure C2. Reaction time (ms) in Experiment 2 depending on Imageability (continuous ratings on a 1-7 Likert scale) and Cue (contextual, irrelevant).

Appendix D: Likert Scales

The following pages include the original German anchors of the Likert scales (and their *English translations*) for the ratings of psycholinguistic variables conducted for the stimuli of Experiment 1 and Experiment 2.

Erregung

gar keine Erregung						sehr starke Erregung
1	2	3	4	5	6	7
○	○	○	○	○	○	○

Arousal

1 = "no arousal at all"

7 = "very strong arousal"

Vorstellbarkeit

sehr schwierig						sehr einfach
1	2	3	4	5	6	7
○	○	○	○	○	○	○

Imageability

1 = "very difficult"

7 = "very easy"

Valenz

sehr negativ | neutral | sehr positiv

-3	-2	-1	0	1	2	3
○	○	○	○	○	○	○

Valence

-3 = "very negative"

3 = "very positive"

0 = "neutral"

Kontext-Verfügbarkeit

sehr schwierig | sehr einfach

1	2	3	4	5	6	7
○	○	○	○	○	○	○

Context availability

1 = "very difficult to think of a context"

7 = "very easy to think of a context"

Emotionales Erleben

sehr wenig | sehr viel

1	2	3	4	5	6	7
○	○	○	○	○	○	○

Emotional Experience

1 = "little emotional experience"

7 = "a lot of emotional experience"

Konkretheit

gar nicht konkret						sehr konkret
1	2	3	4	5	6	7
○	○	○	○	○	○	○

Concreteness

1 = “not concrete at all”

7 = „very concrete”

Ähnlichkeit

gar nicht ähnlich						sehr ähnlich
1	2	3	4	5	6	7
○	○	○	○	○	○	○

Similarity

1 = “not similar at all”

7 = „very similar”

Assoziation

gar nicht assoziiert						sehr stark assoziiert
1	2	3	4	5	6	7
○	○	○	○	○	○	○

Association

1 = “not associated at all”

7 = „strongly associated”

References

1 Spinelli, D. *et al.* Length effect in word naming in reading: role of reading experience and reading deficit in italian readers. *Dev Neuropsychol* **27**, 217-235, doi:10.1207/s15326942dn2702_2 (2005).

2 Brysbaert, M. *et al.* The word frequency effect: a review of recent developments and implications for the choice of frequency estimates in German. *Exp Psychol* **58**, 412-424, doi:10.1027/1618-3169/a000123 (2011).

3 Hoffman, P., Binney, R. J. & Lambon Ralph, M. A. Differing contributions of inferior prefrontal and anterior temporal cortex to concrete and abstract conceptual knowledge. *Cortex; a journal devoted to the study of the nervous system and behavior* **63**, 250-266, doi:10.1016/j.cortex.2014.09.001 (2015).

4 Hoffman, P. The meaning of 'life' and other abstract words: Insights from neuropsychology. *J Neuropsychol* **10**, 317-343, doi:10.1111/jnp.12065 (2016).

5 Borghi, A. M. *et al.* The challenge of abstract concepts. *Psychol Bull* **143**, 263-292, doi:10.1037/bul0000089 (2017).
